# Anaphase Chromosomes in Crane-Fly Spermatocytes Treated With Taxol (Paclitaxel) Accelerate When Their Kinetochore Microtubules Are Cut: Evidence for Spindle Matrix Involvement With Spindle Forces

**DOI:** 10.3389/fcell.2018.00077

**Published:** 2018-07-24

**Authors:** Arthur Forer, Rozhan Sheykhani, Michael W. Berns

**Affiliations:** ^1^Biology Department, York University, North York, ON, Canada; ^2^Beckman Laser Institute and Department of Biomedical Engineering, University of California, Irvine, Irvine, CA, United States; ^3^Department of Bioengineering and Institute for Engineering in Medicine, University of California, San Diego, San Diego, CA, United States

**Keywords:** mitotic mechanisms, spindle matrix, taxol, paclitaxel, laser irradiation, crane-fly spermatocytes

## Abstract

Various experiments have indicated that anaphase chromosomes continue to move after their kinetochore microtubules are severed. The chromosomes move poleward at an accelerated rate after the microtubules are cut but they slow down 1–3 min later and move poleward at near the original speed. There are two published interpretations of chromosome movements with severed kinetochore microtubules. One interpretation is that dynein relocates to the severed microtubule ends and propels them poleward by pushing against non-kinetochore microtubules. The other interpretation is that components of a putative “spindle matrix” normally push kinetochore microtubules poleward and continue to do so after the microtubules are severed from the pole. In this study we distinguish between these interpretations by treating cells with taxol. Taxol eliminates microtubule dynamics, alters spindle microtubule arrangements, and inhibits dynein motor activity *in vivo*. If the dynein interpretation is correct, taxol should interfere with chromosome movements after kinetochore microtubules are severed because it alters the arrangements of spindle microtubules and because it blocks dynein activity. If the “spindle matrix” interpretation is correct, on the other hand, taxol should not interfere with the accelerated movements. Our results support the spindle matrix interpretation: anaphase chromosomes in taxol-treated crane-fly spermatocytes accelerated after their kinetochore microtubules were severed.

## Introduction

Experiments in this report deal with movements of anaphase chromosomes in crane-fly spermatocytes when their kinetochore microtubules are severed. After severing kinetochore microtubules in various cells with either ultraviolet-light microbeam irradiation or visible-light laser microbeam irradiation, the associated anaphase chromosomes continue to move poleward. The movements immediately increase in speed but slow down again some minutes later in most cells (review in Forer et al., 2015). In crane-fly spermatocytes, however, anaphase chromosomes with severed kinetochore microtubules do not increase in speed but rather continue to move with pre-irradiation velocities. This is because the elastic “tethers” that connect separating anaphase chromosomes in crane-fly spermatocytes (LaFountain et al., 2002) coordinate the movements of the separating chromosomes. When the tethers are severed prior to cutting the kinetochore microtubules, the chromosomes act as in other cells: they accelerate for some minutes and then return to their initial velocity (Sheykhani et al., 2017). Experiments in this article deal with the mechanism of continued chromosome movement after kinetochore microtubules are severed.

**Figure 1:**
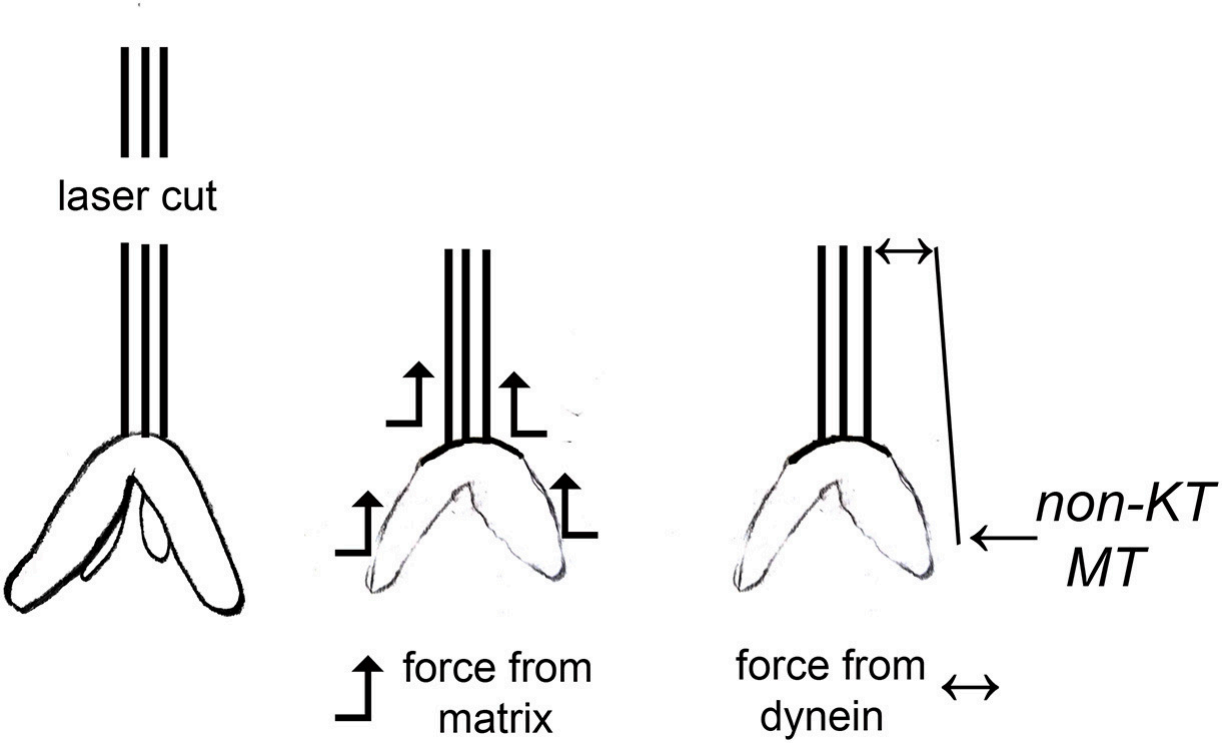
Two different interpretations of movement with severed kinetochore microtubules. Kinetochore microtubules (represented by lines extending from the kinetochore) are cut, leaving only a kinetochore stub attached (leftmost drawing). The two interpretations for how chromosomes move with severed kinetochore microtubules are illustrated: forces from a spindle matrix act on the kinetochore stub and chromosomes to propel them poleward, or dynein at the poleward tip of the kinetochore stub acts against non-kinetochore microtubules to propel the stub (and attached chromosome) poleward. According to the dynein interpretation, if the dynein is inhibited or the non-kinetochore microtubules are not arranged properly, there will be no force and the chromosomes will not move.

There are two published interpretations of why movements continue with severed kinetochore microtubules, as diagrammed in Figure 1. One interpretation is that dynein quickly relocates to the minus ends of the severed microtubules and pushes against neighboring non-kinetochore microtubules to propel the kinetochore microtubule stub poleward (Elting et al., 2014; Sikirzhytski et al., 2014). The other interpretation is that microtubules do not produce the force but rather components of the spindle matrix propel the kinetochore stub microtubules poleward. In this model, forces from the spindle matrix apply poleward forces on kinetochore microtubules and chromosomes during normal anaphase. Kinetochore microtubules must depolymerize before movement occurs and the rate of depolymerization is the rate limiting step for chromosome movement. Severing microtubules eliminates the barrier to faster movement speeds so the chromosomes accelerate when their kinetochore microtubules are severed (e.g., Pickett-Heaps and Forer, 2009; Johansen et al., 2011; Forer et al., 2015). They slow to their normal speed when the kinetochore stub runs into other microtubules.

To distinguish between these two interpretations we treated cells with taxol (paclitaxel), which would be expected to slow or stop chromosome movement with severed kinetochore microtubules if the dynein interpretation is correct, but not if the spindle-matrix interpretation is correct. One reason is that taxol alters the distributions and arrangements of spindle microtubules (e.g., Jordan et al., 1993; Snyder and Mullins, 1993; Rizk et al., 2009). Differently arranged microtubules would inhibit dynein relocation to the severed microtubules and consequently inhibit poleward transport, as noted by Elting et al. (2014). Another reason is that taxol inhibits dynein motor activity, as discussed below. Inhibiting dynein activity would prevent (or slow) chromosome movement with severed microtubules.

A primary effect of taxol is to stabilize microtubules, which occurs when taxol binds to protofilaments. Lower concentrations of taxol prevent addition of tubulin to microtubule (+)-ends, though depolymerization at the minus ends still may occur (Derry et al., 1995; Waters et al., 1996; Yvon et al., 1999; Shannon et al., 2005). At higher concentrations of taxol neither end of the microtubule is active dynamically (e.g., Jordan and Kamath, 2007). Taxol has other effects as well. Taxol alters the configurations of the microtubule protofilaments and of the α- and β-subunits of tubulin (Xiao et al., 2006, 2012; Jordan and Kamath, 2007; Khrapunovich-Baine et al., 2009). Taxol alters binding of MAPs and Tau protein to microtubules and detaches Tau from microtubules (Kar et al., 2003; Samsonov et al., 2004; Xiao et al., 2012). Taxol also inhibits the activities of motor proteins. *In vitro* studies of kinesin and dynein motor activity often use taxol to stabilize microtubules (e.g., Vale et al., 1985; Svoboda et al., 1993; Shah et al., 2000), so on the face of it one would presume that taxol would have no effect on dynein or kinesin activity. However, while dynein seems to be active on taxol-stabilized microtubules *in vitro*, motility due to dynein is indeed inhibited when taxol is applied *in vivo* (e.g., discussed in Gornstein and Schwarz, 2014). Several studies report, for example, that taxol inhibits the dynein-dependent *in vivo* transport of cargo along cytoplasmic microtubules (Kristensson et al., 1986; Lin and Collins, 1992; Nakata and Yorifuji, 1999; Giannakakou et al., 2000; Shemesh and Spira, 2010) and inhibits dynein-dependent movements of cargo to nuclei along microtubules (Suikkanen et al., 2003; Hirosue et al., 2007; Li et al., 2012; Pawlica et al., 2014; Bai et al., 2017). The dynein interpretation of movement with severed microtubules requires that dynein be active, so inhibition of dynein by taxol should impede chromosomes from moving. If chromosomes in taxol-treated cells continue to move (and accelerate) this would indicate that the motile forces arise from something other than dynein. Since taxol would not affect actin, myosin or spindle matrix proteins, these could be the producers of the motile forces that move the chromosomes. Thus, we treated crane-fly spermatocytes with taxol.

In crane-fly spermatocytes, taxol at concentrations as low as 5 nM prevents addition of tubulin subunits to kinetochore microtubules at the kinetochore (+)-ends, as indicated by the kinetochore ends of the microtubules becoming acetylated (Wilson and Forer, 1997). Taxol at nanomolar concentrations causes kinetochore microtubule bundles to become thinner, stabilizes non-kinetochore microtubules, which become acetylated, and causes spindles to change shape and microtubules to have altered arrangements (Wilson and Forer, 1997). Treatment of entire crane-fly testes with 5–10 μM taxol causes spermatocyte spindles to change shape and spindle microtubules to rearrange but does not block anaphase onset (LaFountain et al., 2001); however, anaphase movements are slowed considerably, from average speeds of 0.5 μm/min in control cells to average speeds of 0.1 μm/min in taxol-treated cells (LaFountain et al., 2001). In those experiments LaFountain et al. (2001) also showed that taxol interferes with normal spindle transport mechanisms: in control cells, akinetic arm fragments produced in metaphase (by severing arms) move poleward at the same speeds as anaphase chromosomes, but in taxol-treated cells the arm fragments do not move at all. Thus, taxol eliminates the usual transport properties of crane-fly spermatocyte spindles.

In our experiments nanomolar concentrations of taxol were added directly to crane-fly spermatocytes and we monitored whether chromosomes accelerated after their kinetochore fibers were cut with a laser microbeam.

## Methods

Crane-fly spermatocytes were obtained by cutting open larvae under halocarbon oil, removing the testes, rinsing off the oil with insect Ringers solution, and breaking the testes open into a 2.5 μl drop of insect Ringers solution (on a coverslip). The Ringers solution contained fibrinogen. We added thrombin to cause the fibrinogen to clot, and then placed the coverslip in a perfusion chamber and perfused the preparation with insect Ringers solution, as described in Forer and Pickett-Heaps (2005) and Sheykhani et al. (2017). Cells then were perfused with insect Ringers solution that contained either 10, 15, or 20 nM taxol. The experiments reported herein are of cells immersed in taxol for at least 20 min. before starting the laser experiments. Taxol was obtained from LC Laboratories.

Cells were observed with a phase-contrast Zeiss Plan-Apochromat 63x NA 1.40 objective, and digital images were recorded every 3–4 s. The images were time-lapsed and further analyzed to obtain distance-vs.-time graphs of chromosome movement, as described in Sheykhani et al. (2017). Briefly, individual bmp images were observed. For each image we marked a fixed position at the equator or at a pole. We specified the time the image was recorded (as embedded in the image) and marked the position of the object(s) in question (e.g., kinetochores and/or telomeres). A computer program converted the data from each image into distance from the fixed point vs. time. We obtained velocities as the slopes of the distance-vs.-time graphs. The laser irradiation methods were as described in Sheykhani et al. (2017). In brief, we recorded cells and irradiated regions of interest using a 740 nm wavelength 200-fs pulsed laser in the microscope system described in Shi et al. (2012) and Harsono et al. (2013). Irradiations were usually in three different planes of focus separated in the Z-axis by 0.4 μm.

Some irradiated cells were stained using anti-tubulin antibodies and studied using confocal immunofluorescence microscopy as described in Sheykhani et al. (2017). Briefly, irradiated cells were lysed in microtubule-stabilizing buffer with added detergents, fixed with 0.2% glutaraldehyde, treated with sodium borohydride, and rinsed in PBS. Subsequently cells were reacted first with anti-tubulin antibody YL1/2 and then secondary antibody bound to Alexa 488 or Alexa 594 fluorophores. The stained specimens were observed using an Olympus Fluoview 300 confocal microscope.

## Results

**Figure 2:**
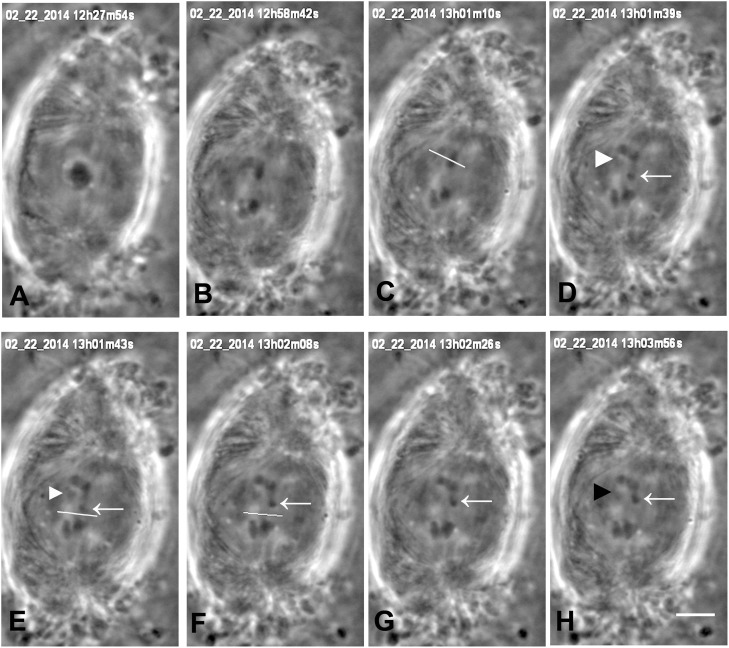
Cutting arms and tethers in anaphase, in a cell treated with 15 nM taxol. The 3 bivalent chromosomes were in different focal planes; the images in Figure 2 are in a plane of one of the chromosomes. The date and time of each image is at the top of the frame. **(A)** the cell is in metaphase. One bivalent is seen at the equator. **(B–H)** anaphase. Two arms are severed from the half-bivalent moving to the upper pole. The white line in **(C)** indicates the position of the laser cut. The arrowhead and arrow (in **D–H**) indicate the two arm fragments produced by the cut. The arm fragment on the right (arrow) moves toward toward the partner arm moving to the opposite pole while the arm fragment on the left (arrowhead) does not move. [In crane-fly spermatocytes, each separating pair of chromosomes is connected by tethers, but there are tethers between only two of the four arms (LaFountain et al., 2002; Sheykhani et al., 2017)]. The tethers between the separating chromosomes were cut, as indicated by the white line in **(E)**, before the cut, and in **(F)**, after the cut. The arm fragment moved back toward its original chromosome after the tether was cut, as sometimes happens in control (not-treated) cells (Sheykhani et al., 2017); this presumably is because the arm was incompletely severed and a piece remained with weaker elasticity than the tether, but strong enough to move the arm fragment back again after the tether was cut. The scale bar in **(H)** represents 5 μm.

In the presence of 10–20 nM taxol crane-fly spermatocyte spindles often have altered shapes: the poles become broadened, the spindles often are short and squat, sometimes resembling barrels (Wilson and Forer, 1997; LaFountain et al., 2001), similar to spindles in other cells (e.g., Snyder and Mullins, 1993). Crane-fly spermatocytes enter anaphase in the presence of 10–20 nM taxol (e.g., Figure 2), as described by Wilson and Forer (1997) and LaFountain et al. (2001), and generally complete anaphase. Poleward velocities of chromosomes in our taxol-treated cells averaged 0.18 μm/min, considerably slower than average velocities in control cells (~0.5 μm/min; LaFountain et al., 2002; Sheykhani et al., 2017).

**Figure 3:**
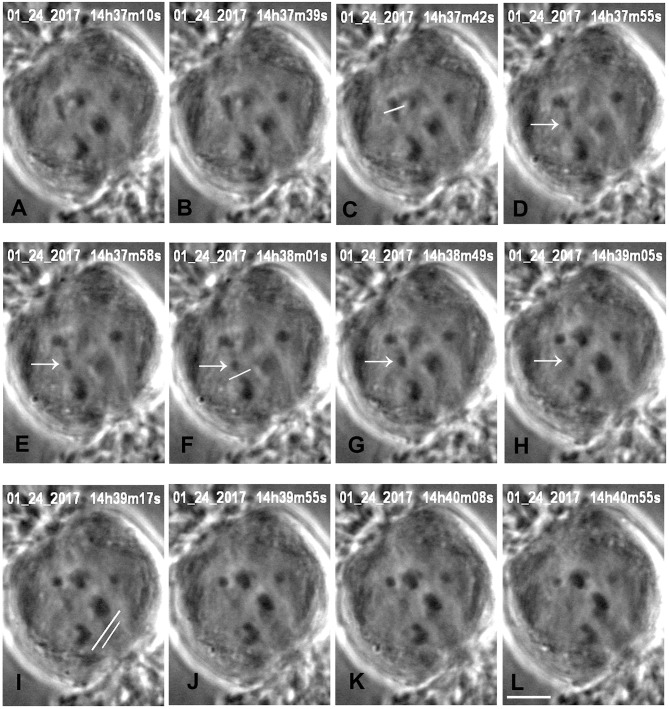
Cutting an arm and its tether in anaphase, in a cell treated with 15 nM taxol. All images are at successive times of anaphase, at times indicated at the top of each image **(A,B)**, before severing an arm. One arm from the upper left half-bivalent is severed at the position indicated by the white line in **(C)**. The arrow (in **D–H**) points to the arm fragment produced, and the white line in **(F)** indicates the position of the cut when the tether was severed. The arm fragment ceased backward movement after its tether was cut and remained in a more-or-less constant position. The image in **(I)** shows the positions of the laser cuts (white lines) just before the kinetochore fiber regions were cut for both chromosomes moving to the lower pole. The left chromosome (tethers cut) accelerated after the kinetochore fibers were cut **(J–L)**, the right chromosome (tethers not cut) did not. The scale bar in **(L)** represents 5 μm.

**Figure 4:**
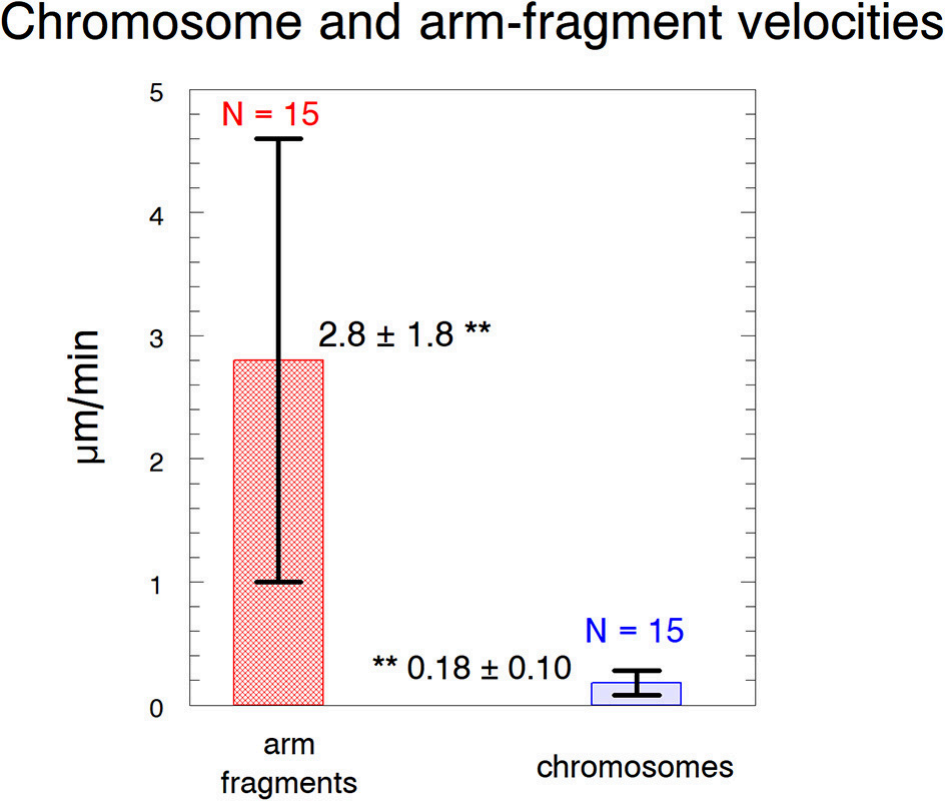
Velocities of arm fragments and chromosomes. The velocities are of arm fragments produced in anaphase compared to the anaphase velocities of the same chromosomes. Error bars represent standard deviations. Arm fragment velocities (2.8 ± 1.8 μm/min) are statistically significantly different (^**^) from chromosome velocities (0.18 ± 0.10 μm/min) at *p* < 0.00001 using Students *t*-test. N, number measured. The data are from 6 cells treated with 10 nM taxol and 9 cells treated with 15 nM taxol.

We produced arm fragments by severing terminal portions of chromosome arms during anaphase. This was part of our experimental protocol (described below). Arm fragments produced in anaphase taxol-treated cells behave as they do in not-treated cells: they move backwards across the equator toward their partner chromosomes moving to the opposite pole (Figures 2, 3), as described previously in crane-fly spermatocytes (LaFountain et al., 2002; Sheykhani et al., 2017) and other animals cells (Forer et al., 2017). In our experiments arm-fragment velocities in taxol-treated cells were considerably faster than the velocities of the associated chromosomes (Figure 4), as previously reported in control cells (LaFountain et al., 2002; Sheykhani et al., 2017).

**Figure 5:**
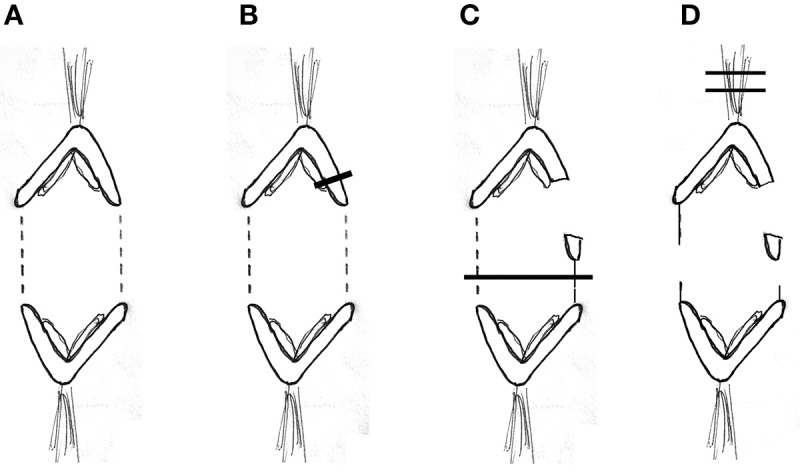
Diagrammatic illustration of the experimental procedure. One pair of separating anaphase chromosomes is illustrated. Tethers between the two connected arms are represented by dashed lines **(A)**. The laser cutting the upper right arm is indicated by the dark line **(B)**. The arm fragment moves toward the partner **(C)**, which also illustrates the laser line (dark horizontal line) cutting the contracted tether (solid vertical line) and the tether between the left-most pair of arms. The arm fragment stops moving when the tether is cut **(D)**, which also illustrates the laser lines (dark horizontal lines) cutting the upper kinetochore fiber.

**Figure 6:**
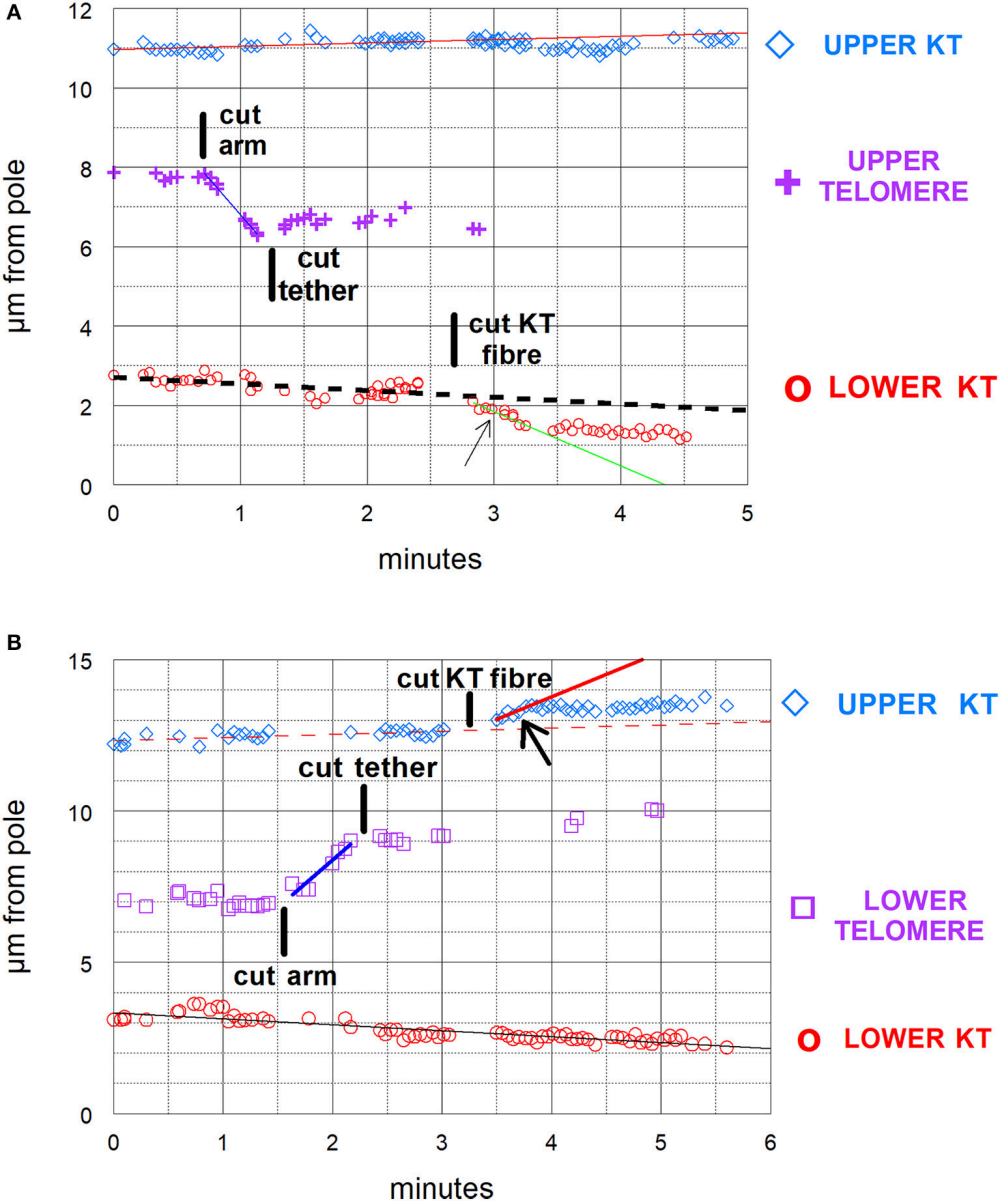
**(A,B)** Graphical illustrations of chromosome acceleration after cutting tethers. Both distance-vs-time graphs are of cells in which an arm was cut, followed by cutting its tether, followed by cutting a kinetochore spindle fiber (KT fiber). The graphs include the positions of the separating kinetochores (KT) and of the telomeres of the cut arm, all as measured vs. one of the spindle poles. The lines on the graphs represent the lines of best fit to the corresponding points. **(A)** is the same cell illustrated in Figure 3 and shown in Supplementary Video 1. In **(A)** the kinetochore fiber to the bottom pole was cut and in **(B)** that to the top pole was cut. The kinetochores with cut kinetochore fiber accelerated after the cuts: the higher speed motion lasted for about 35–40 s in **(A)** and for under 30 s in **(B)**. In **(A)** the velocity of the upper chromosome (upper KT) was 0.09 μm/min; the lower chromosome velocity prior to cutting its KT-fiber was 0.17 μm/min and during the accelerated movements after cutting its KT fiber was 1.3 μm/min. The velocity of the arm fragment (before cutting its tether) was 3.7 μm/min. In **(B)** the velocity of the lower chromosome was 0.2 μm/min. That of the upper chromosome prior to cutting its KT fiber was 0.1 μm/min and that in the accelerated movements after cutting its KT fiber was 1.5 μm/min. The velocity of the arm fragment before its tether was cut was 3.1 μm/min.

**Figure 7:**
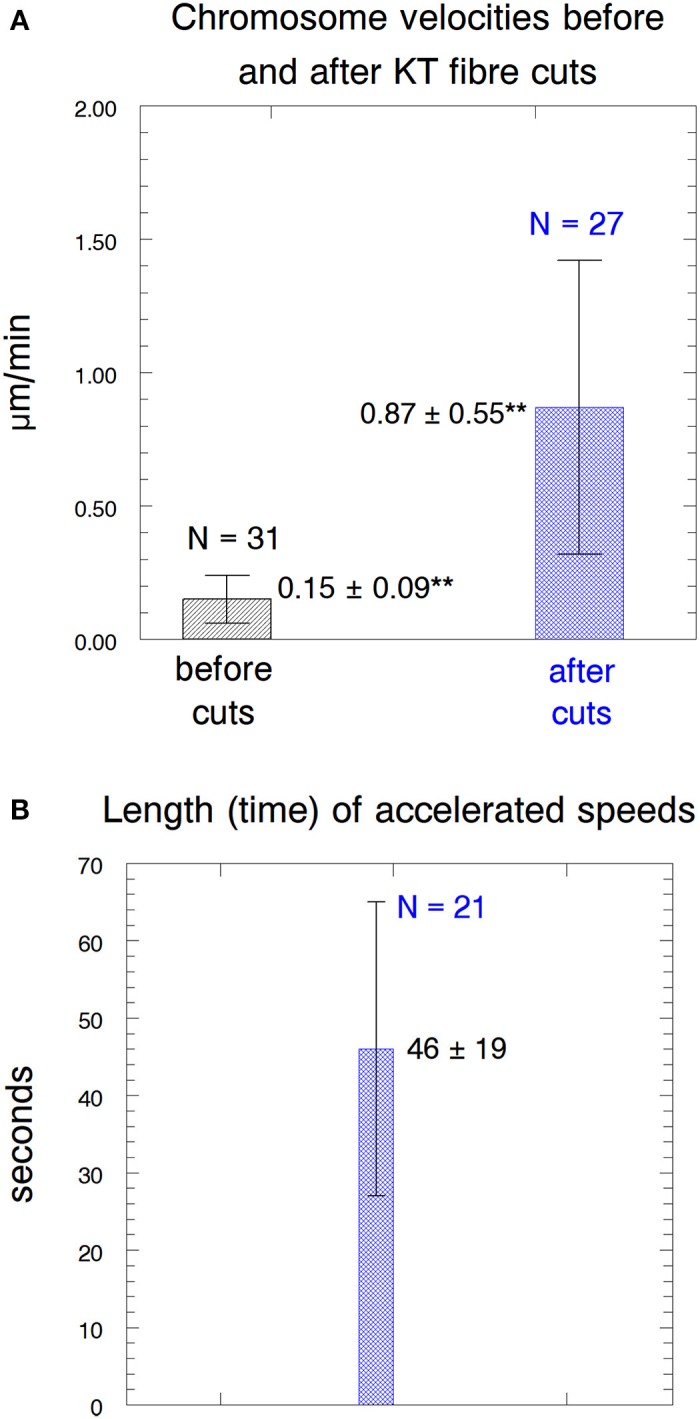
Parameters of the accelerated motion. **(A)** Chromosome velocities before and after acceleration. The error bars represent standard deviations. The velocities before cutting kinetochore fibers (0.15 ± 0.09 μm/min) were statistically significantly different (^**^) from those after kinetochore fibers were cut (0.87 ± 0.55 μm/min) at *p* < 0.00001, using Students *t*-test. *N*, number of observations. **(B)** The length of time that the accelerated speeds lasted. N, number of observations.

Our experimental design to cause anaphase chromosome acceleration was the same that Sheykhani et al. (2017) used to induce chromosome acceleration in not-treated spermatocytes. Our procedures are shown in Figure 5, in which 1 anaphase chromosome and its partner are illustrated, moving to opposite poles. Dashed lines (representing tethers) extend between the two pairs of arms that are connected by tethers (Figure 5A). We first cut a portion of an arm (Figure 5B). As the resultant arm fragment moved backward toward its partner, its tether was severed by cutting across the region between the two telomeres (Figure 5C). If the fragment stopped moving (Figures 2, 3) that indicated that its tether was cut. To ensure that both tethers associated with that chromosome are cut, the laser was positioned to cut across the width of the chromosomes in question (Figure 5C; also Figures 2, 3), and cuts were done in 3 different Z-planes. After cutting the tethers we cut the associated kinetochore fibers (Figures 3, 5D), which caused the associated chromosomes to speed up (Figures 6A,B,7A). After less than a minute (on average) the chromosomes returned to their original velocities (Figure 7B). Supplementary Videos 1, 2 show two different cells treated this way. The values in Figure 7 include treatments with 10 or 15 nM taxol lumped together because there were no significant differences between these concentrations (at *p* < 0.05, using Students *t*-test) in anaphase velocities, or length of time during which the speed was increased. Chromosome movements were so slow in taxol-treated cells that in two separate cells we were able to cut the same kinetochore fiber twice. In both cells the kinetochore fibers were irradiated for a second time after the chromosome slowed down to near its original speed and in both cells the chromosome accelerated again after the second cut.

**Figure 8:**
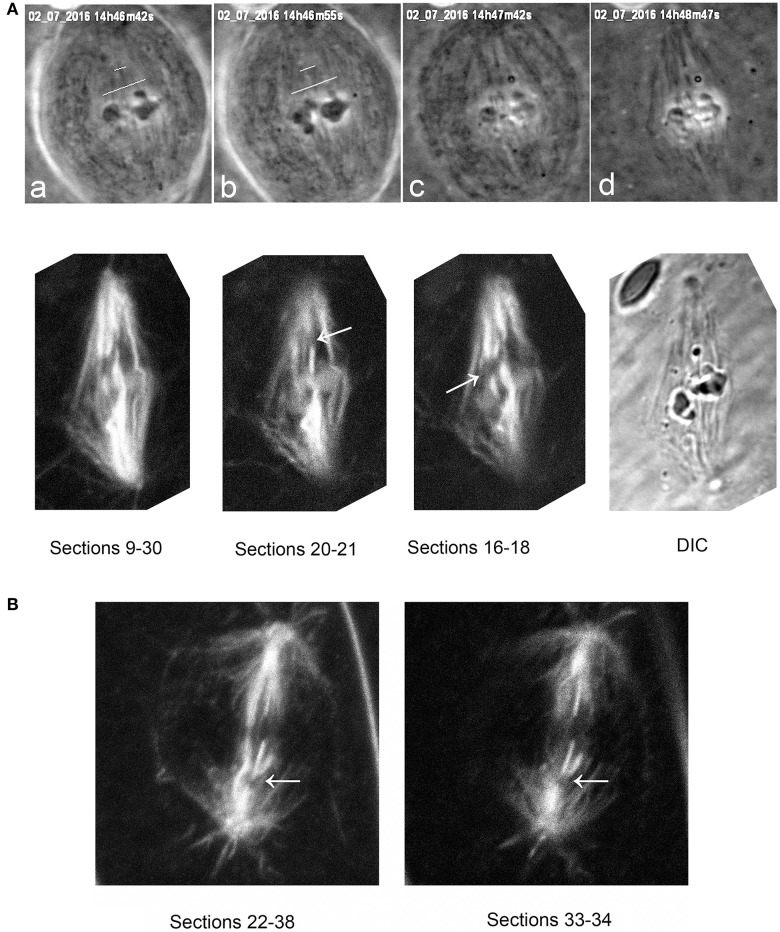
**(A)** Tubulin staining after cuts of spindle fibers, illustrating that the laser severs taxol-stabilized microtubules. The top row illustrates phase-contrast images of a metaphase cell during and after the cuts, and the second row the same cell after staining for tubulin. The top row shows the metaphase cell viewed in phase-contrast microscopy **(a)** prior to cutting (at the white lines), **(b)** just after cutting, **(c)** as lysis started, and **(d)** after lysis was complete. The cut microtubules are visible in **(c,d)**. The second row shows the same cell studied in the confocal microscope, after summing the Z-series of images through most of the sections (sections 9–30), and then through smaller groups of sections (20–21 and 16–18), showing that the laser severed the taxol-stabilized spindle microtubules. The arrows point to the cut kinetochore microtubule regions. **(B)** a taxol-stabilized spindle cut after lysis. The arrow indicates the region of the cut, illustrating that the laser severs taxol-stabilized microtubules.

It seemed clear that the laser severed the microtubules, for otherwise the chromosomes would not accelerate when there are taxol-stabilized microtubules in their way. (Supplementary Video 1 illustrates a cell in which kinetochore fibers of two chromosomes were cut, but only the chromosome with severed tethers accelerated.) To verify that the laser indeed cut the kinetochore microtubules we studied anti-tubulin fluorescence in taxol-treated cells in which the laser was used to cut spindles. Confocal microscope images of the cut regions indicate that the kinetochore microtubules indeed were cut (Figure 8), as they are in not-treated cells (Sheykhani et al., 2017).

Since the experimental protocol used for taxol-treated cells was the same as for not-treated cells (Sheykhani et al., 2017), and the same result was obtained, namely that chromosomes moved faster when their kinetochore fibers were cut, we conclude that taxol treatment does not prevent the associated chromosome from accelerating when its kinetochore microtubules are severed.

## Discussion

In the presence of 10–15 nM taxol, the slowly moving anaphase chromosomes (in crane-fly spermatocytes) accelerated after their tethers first were severed and then their kinetochore microtubules were severed. As discussed in the Introduction, taxol blocks microtubule dynamics, alters the distributions of spindle microtubules, blocks motor activity of dynein and kinesin when applied *in vivo*, and (in crane-fly spermatocytes) blocks akinetic chromosome transport. Since blocking dynein and rearranging microtubules would stop the accelerated movements under the interpretation that the continued movement is due to dynein (Figure 1), our data support the interpretation that chromosomes move with severed microtubules because of forces from the spindle matrix and/or actin-myosin. A variety of other data support this proposition, summarized for example in Pickett-Heaps and Forer (2009), Johansen et al. (2011), and Forer et al. (2015). In brief, the presence of a spindle matrix was deduced from physiological experiments, and putative matrix proteins were identified as a set of proteins that kept the shape of a spindle after spindle microtubules were rapidly depolymerized (Johansen and Johansen, 2007; Johansen et al., 2011). One of these, *skeletor*, is implicated in control of spindle function from experiments in which anti-skeletor antibodies were injected into *Drosophila* embryos (Johansen et al., 1996; Walker et al., 2000). Another, *chromator*, is implicated in spindle function in that chromosome movement was perturbed after either depletion of chromator by RNAi or by direct mutation of the chromator gene (Rath et al., 2004; Ding et al., 2009). Myosin and actin involvement in force production, and perhaps association with the spindle matrix (Johansen et al., 2011), is indicated by a variety of experiments including both pharmacological and molecular genetic approaches (discussed in Pickett-Heaps and Forer, 2009; Sheykhani et al., 2013; Mogessie and Schuh, 2017). It still is not clear what the spindle matrix is composed of, how it acts, and how actin and myosin might interact with chromator or other matrix components (e.g., Johansen et al., 2011), so it is difficult to know how the forces from the spindle matrix are produced.

In the original work that led to the spindle matrix hypothesis, chromosomes in diatom spindles moved poleward after colchicine removed kinetochore microtubules (e.g., Pickett-Heaps and Spurck, 1982, reviewed in Pickett-Heaps and Forer, 2009). In these experiments there is no ambiguity about possible roles for microtubules in producing the force because the chromosomes moved only in the absence of microtubules. The same result was obtained in recent experiments using spermatocytes of the flatworm *Mesostoma*: chromosomes moved poleward at high speeds after nocodazole depolymerized the spindle microtubules (Fegaras and Forer, 2018). These observations further support the hypothesis that motile forces are produced by non-microtubule components such as actin-myosin or spindle matrix proteins.

The speeds of the accelerated chromosome movements in taxol-treated cells (after severing kinetochore microtubules) also suggest that the forces for chromosome movement arise from components other than microtubules. Anaphase velocities were much faster in control cells than in taxol-treated cells but the velocities of the accelerated chromosomes were the same (Table 1). That is, while anaphase velocities differed by more than a factor of 2.5 between control and taxol-treated cells, anaphase movements with severed microtubules were at the same speeds (*p* = 0.4–0.5, using Students *t*-test). This indicates that the accelerated speeds are due to a non-microtubule force producer.

**Table 1:** Normal and accelerated anaphase chromosome velocities.

	**Normal chromosome velocities**	**Accelerated chromosome velocities**
Control cells	0.5 μm/min LaFountain et al., 2001; Sheykhani et al., 2017	0.96 μm/min^**^ Sheykhani et al., 2017
Taxol-treated cells	0.18 μm/min (Figure 4)	0.87 μm/min^**^ (Figure 7A)
		

** *p = 0.4–0.5 using Students t-test*.

A clear difference between accelerated movements in taxol-treated cells vs. in not-treated cells is the length of time that the increased speeds last before the chromosomes slowed down again. In control (not-treated) cells the time before slowdown averaged 125 s (Sheykhani et al., 2017) whereas in taxol-treated cells it was 46 s (Figure 7), a significant difference (*p* < 0.00001, using Student′s *t*-test). The reason for chromosome acceleration and subsequent speed reduction was not considered in proposing that dynein causes the movement (Elting et al., 2014). On the other hand, acceleration and slowdown are inherent in the proposal that the spindle matrix causes the movement: movement is faster because severing the microtubules removes the barrier that slows down movement, and the movement slows down again when the kinetochore stub encounters obstacles such as other microtubules extending from the poles. The observation that taxol treatment of crane-fly spermatocytes produces increased numbers of interpolar microtubules (Wilson and Forer, 1997) and produces greatly increased numbers and densities of short microtubules that extend from the poles (LaFountain et al., 2001) fits our interpretation by indicating that the kinetochore stub would encounter barriers in a shorter time. Further, the observation observation that chromosomes accelerated a second time after the kinetochore fiber is cut a second time also suggests that microtubules intervening between kinetochore stub and pole cause the accelerated chromosomes to slow down. Kinetochore stubs formed in anaphase control cells elongate as anaphase progresses (e.g., Pickett-Heaps et al., 1996; Spurck et al., 1997), but they are unlikely to do so in taxol-treated cells in which microtubule dynamics are inhibited. Thus, the second cut of the “kinetochore fiber” most likely severs the intervening microtubules, and the second acceleration indicates that the initial slowing down of chromosomes is due to the intervening microtubules. These observations, too, suggest that poleward forces act on kinetochore microtubules, and are not produced by them.

Aspects of our experiments peripheral to how force is produced with severed kinetochore microtubules deal with further evidence that elastic “tethers” cause the movements of arm fragments (cut from telomere-containing ends of anaphase chromosomes) toward their separating partner chromosomes (across the equator). The experimental evidence that these movements are due to elastic tethers that extend between chromosomes and not to interzonal microtubules (discussed in LaFountain et al., 2002; Forer et al., 2017; Paliulis and Forer, 2018) basically is that arm-fragment movement stops after ablation of either the moving telomere or the partner′s telomere (to which the fragment is moving). Our results on movements of arm fragments in taxol-treated cells add to this argument because taxol-treatment eliminates the transport properties of crane-fly spermatocyte spindle microtubules: akinetic pieces of chromosomes in not- treated crane-fly spermatocytes move poleward, but akinetic pieces in taxol-treated spermatocytes do not move poleward, showing that the non-dynamic spindle microtubules in taxol-treated cells no longer support their movement (LaFountain et al., 2001). In our experiments reported here, telomere-containing arm fragments produced in taxol-treated anaphase cells move across the equator to opposite telomeres at about the same speed as in non-treated cells (2.8 μm/min, Figure 4, vs. 4.8 μm/min, Sheykhani et al., 2017, *p* = 0.1–0.2 using Students *t*-test). Thus, the movements of arm fragments are not due to microtubules and, consistent with previous evidence (LaFountain et al., 2002), are rather due to elastic forces produced by “tethers” that extend between separating telomeres.

In summary, our data show that anaphase chromosomes move much slower than normal in the presence of taxol and that, as in not-treated cells, they accelerate when their kinetochore microtubules are severed (after first severing the tethers that connect separating partner chromosomes). The accelerated speeds in the taxol-treated cells are the same as those in not-treated cells. Since taxol blocks *in vivo* motility due to dynein, and since the accelerated movements are at the same speeds as in cells not-treated with taxol, our experiments suggest that anaphase forces act on microtubules and are not produced by them. They argue against the hypothesis that movements with severed kinetochore microtubules arise from dynein activity and rather support the hypothesis that the movements are due to non-microtubule components associated with the spindle matrix.

## Author contributions

AF used the equipment in MB's laboratory at University of California at San Diego to do the laser-cutting experiments. RS and AF did the confocal microscope work. MB collaborated on the experiments as they were being done. AF and MB did most of the writing and editing, and RS contributed to the editing.

### Conflict of interest statement

The authors declare that the research was conducted in the absence of any commercial or financial relationships that could be construed as a potential conflict of interest.

## Supplementary material

Supplementary Video 1 The same cell illustrated in the montage of Figure 3 and the graph of Figure 6A. The red line starting at 14:37:42 indicates when the laser was on, cutting the arm. The red line starting at 14:38:01 indicates when the laser was on cutting tethers of the arm fragment and the other arm, repeating at 14:38:36. The red lines starting at 14:39:20 indicate when the laser was on cutting the kinetochore fibers of both chromosomes moving to the bottom pole.

Supplementary Video 2 The red line starting at 11:57:11 indicates the laser is on cutting 2 arms from the upper right chromosome. The red lines starting at 11:57:44 indicate when the laser is on cutting the tethers associated with that chromosome. The red lines starting 11:59:07 indicate when the laser is on cutting the kinetochore fibers of the partner chromosome (lower right) to the chromosomes whose arms were cut.
